# Supplementary material for: Integration of Single-Cell Analysis and Bulk RNA Sequencing Data Using Multi-Level Attention Graph Neural Network for Precise Prognostic Stratification in Thyroid Cancer
Source: Cancers (Basel). 2025 Jul 21;17(14):2411. doi: 10.3390/cancers17142411 (PMC12294065; doi:10.3390/cancers17142411)
Supplement: Supplementary file 1 [file cancers-17-02411-s001.zip › cancers-3661084-supplementary.pdf]

Supplementary Material

Unraveling the Role of T Cell in Thyroid Cancer Prognosis through Single Cell Analysis and Multi-level Attention Graph Neural Network Approach

Supplementary Figure S1. 15 scRNA-seq samples of thyroid cancer into distinct clusters. 2

Supplementary Figure S2. Sub-grouping based on specific criteria to analyze T cells landscape. 3

Supplementary Figure S3. Volcano plot showing differential expression between T cells of tumor versus normal samples in thyroid cancer scRNA-seq data. Dashed lines indicate false discovery rate (FDR) of 0.1 and log2 fold change (logFC) of 0.5. 4

Supplementary Figure S4. Analysis of metabolism pathways in in tumor tissues and normal tissues. 5

Supplementary Figure S5. Intercellular communication comparison between tumor tissues and normal tissues ....6.

Supplementary Figure S6. Analysis of Bulk RNA expression patterns in thyroid cancer of TCGA. 7

Supplementary Figure S7. Correlation analysis to assess the association between risk scores and clinical characteristics 8

Supplementary Figure S8. Correlation analysis to assess the association between risk scores and different stages group. 9

Supplementary Figure S9. Correlation analysis to assess the association between risk scores and different T&N stages group. 10

Supplementary Figure S1. 15 scRNA-seq samples of thyroid cancer into distinct clusters.

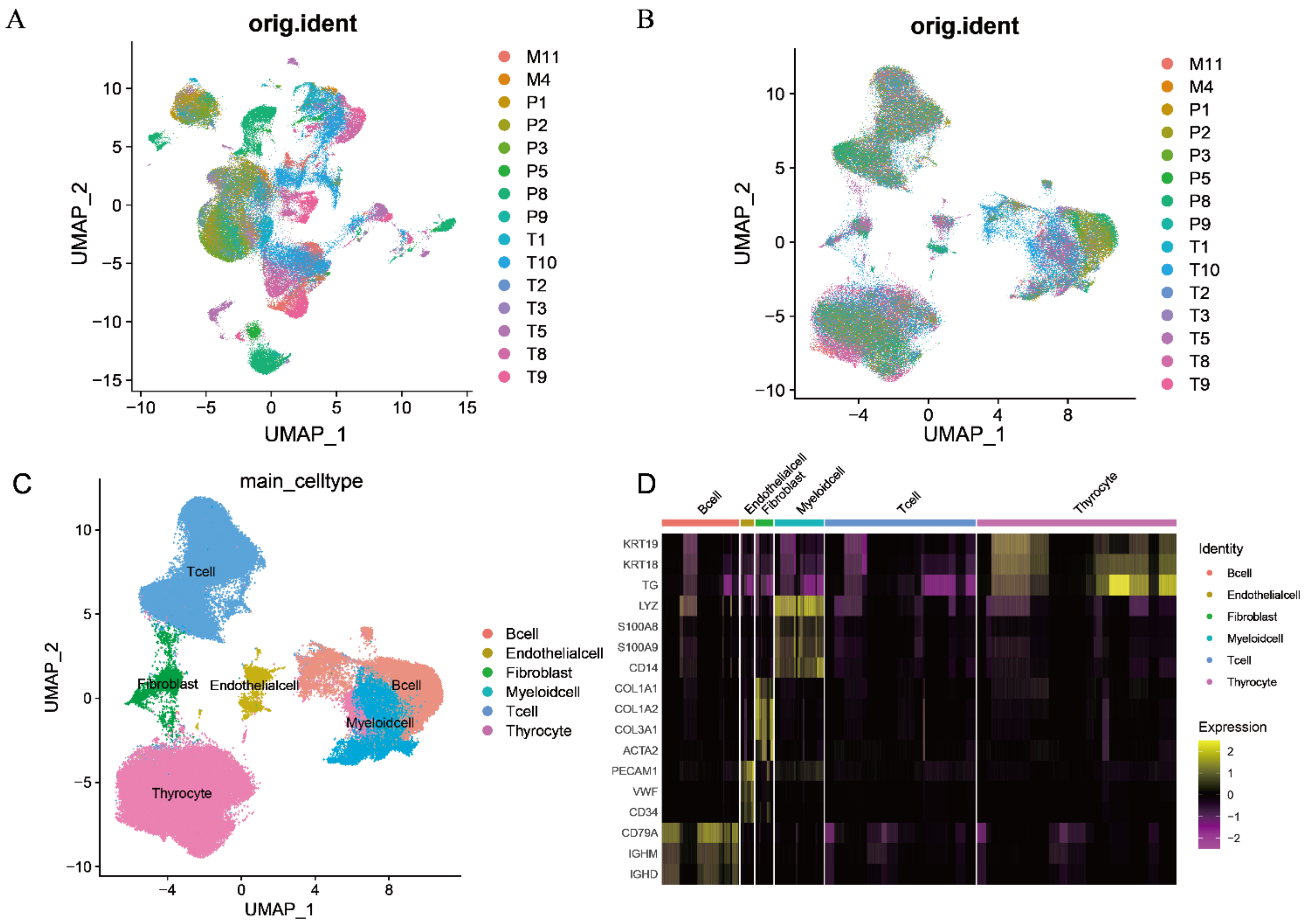

(A-B) UMAP plots showing the distribution of cells from 15 single-cell RNA-seq samples. (C) UMAP plot for the cell type identification. (D) Heatmap showing marker genes for 6 distinct cell types.

Supplementary Figure S2. Sub-grouping based on specific criteria to analyze T cells landscape.

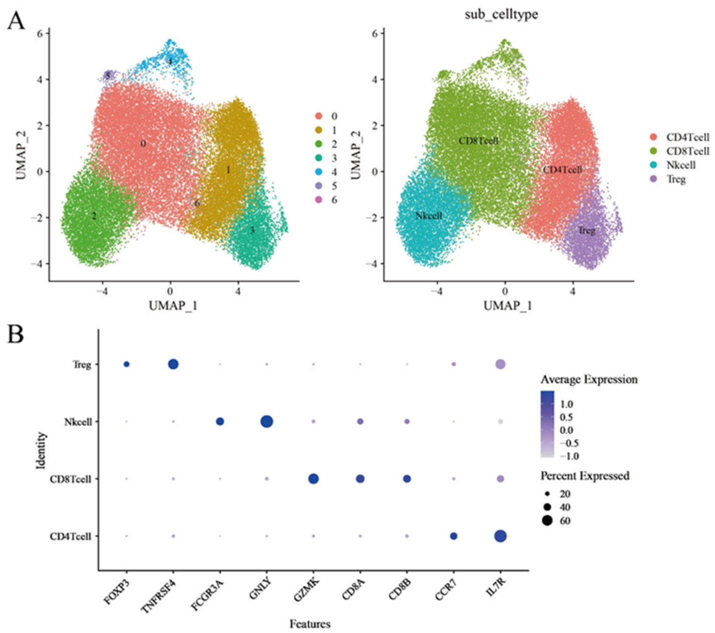

(A) UMAP plots for T cells identification of 36,443 high-quality single cells and annotated with the indicated cell type labels. (B) DotPlot was generated to visualize the expression level of T cells sub-group markers in the specified cell subsets.

Supplementary Figure S3. Volcano plot showing differential expression between T cells of tumor versus normal samples in thyroid cancer scRNA-seq data. Dashed lines indicate false discovery rate (FDR) of 0.1 and log2 fold change (logFC) of 0.5.

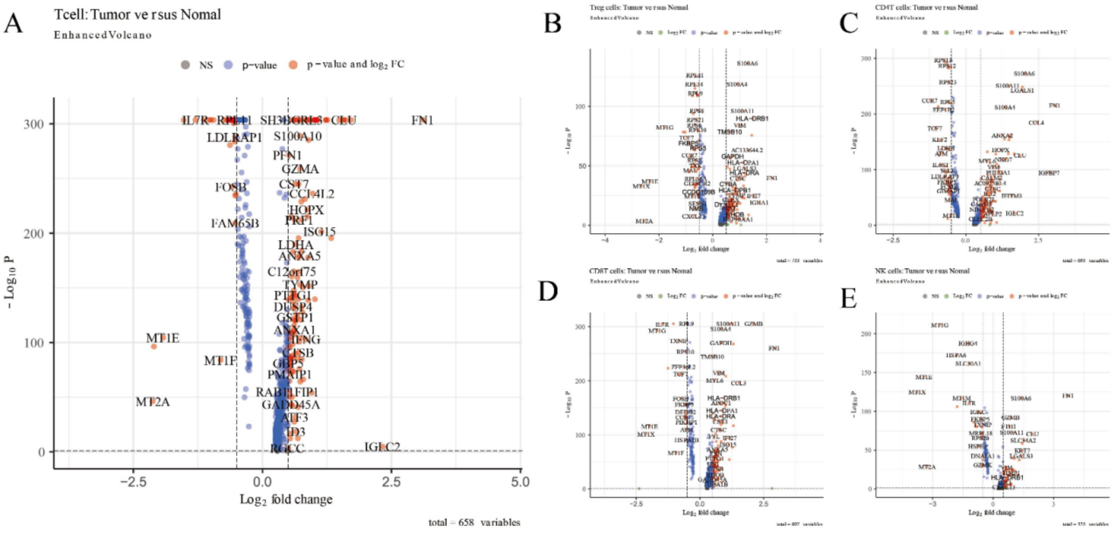

(A) T cells. (B) Treg cells. (C) CD4+ T cells. (D) CD8+ T cells. (E) NK cells.

Supplementary Figure S4. Analysis of metabolism pathways in in tumor tissues and normal tissues.

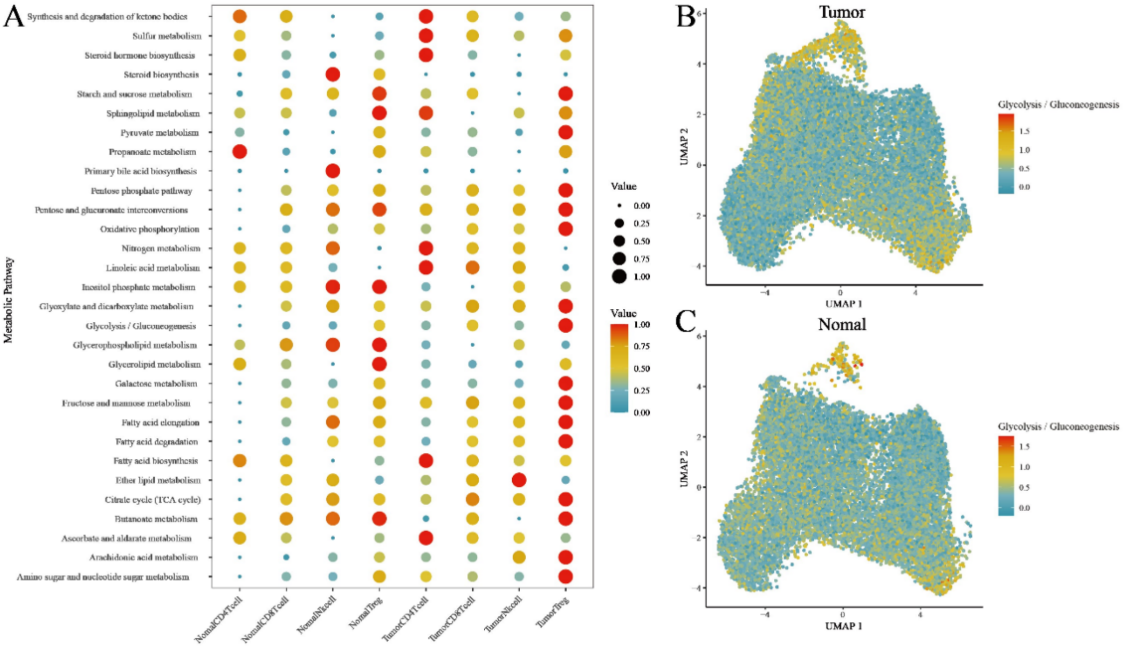

(A) The differences of T cells sub-group in all metabolic pathway activity between tumor tissues and normal tissues. Selections were made by considering evidence from existing literature and ensuring biological interpretability. (B-C) UMAP plots for the expression level of Glycolysis / Gluconeogenesis in tumor tissues and normal tissues.

Supplementary Figure S5. Intercellular communication comparison between tumor tissues and normal tissues

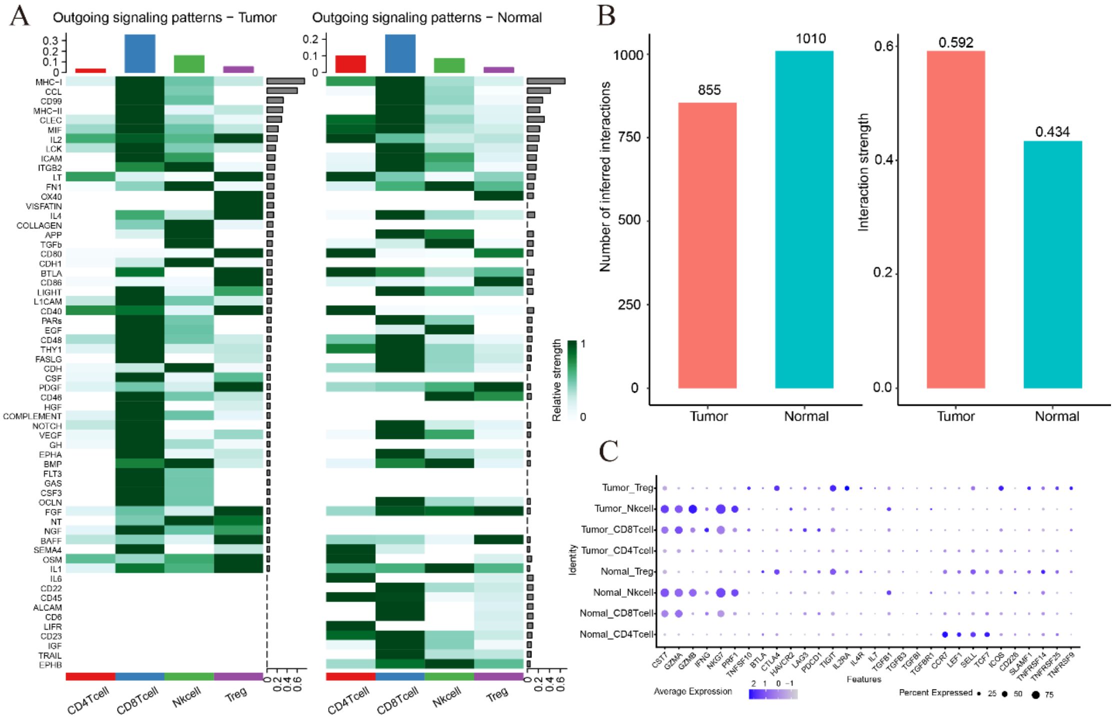

(A) Heatmap of outgoing signaling patterns across immune cell subtypes (CD8$^+$ T cells, CD4$^+$ T cells, NK cells, and Treg cells) in tumor (left) and normal (right) tissues. (B) Bar plots comparing the total number of inferred interactions (left) and the average interaction strength (right) between tumor and normal tissues. (C) Dot plot showing the expression of selected ligand or receptor genes involved in key immune-related signaling pathways across tumor and normal tissues.

Supplementary Figure S6. Analysis of Bulk RNA expression patterns in thyroid cancer of TCGA.

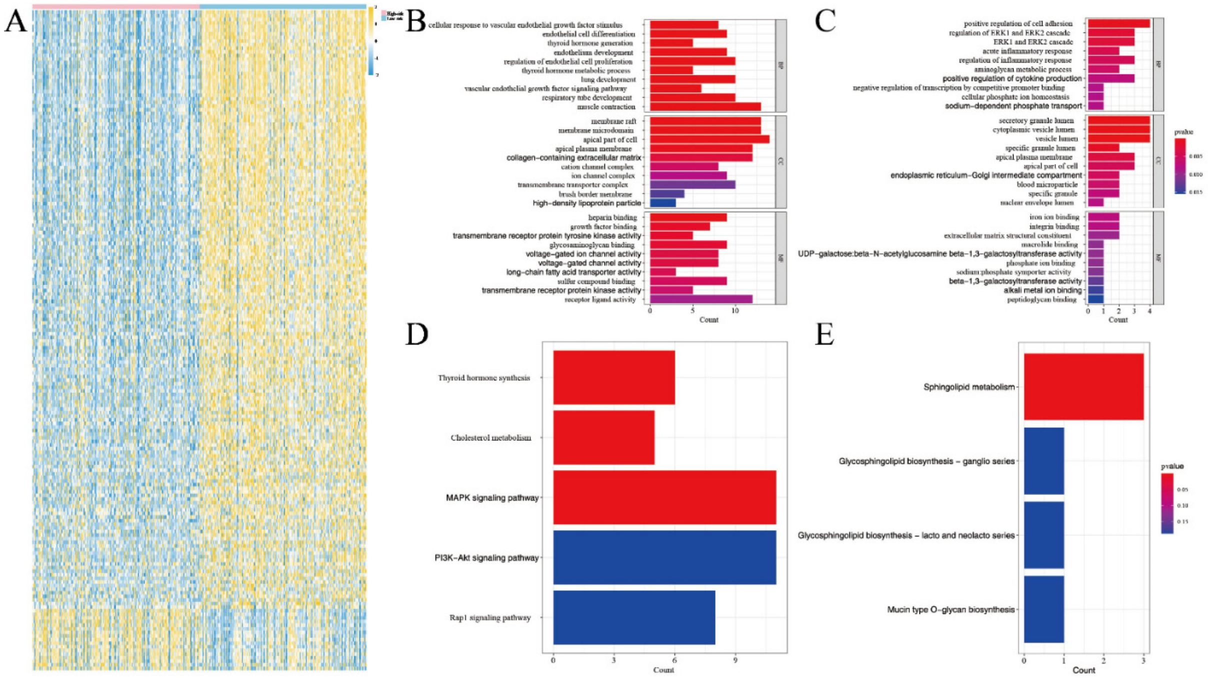

(A)A heatmap showing the differences expression genes in the high-risk and low-risk groups. (B-C) GO enrichment analysis of the high-risk and low-risk groups. (D-E) KEGG pathway enrichment analysis of the high-risk and low-risk groups.

Supplementary Figure S7 Correlation analysis to assess the association between risk scores and clinical characteristics.

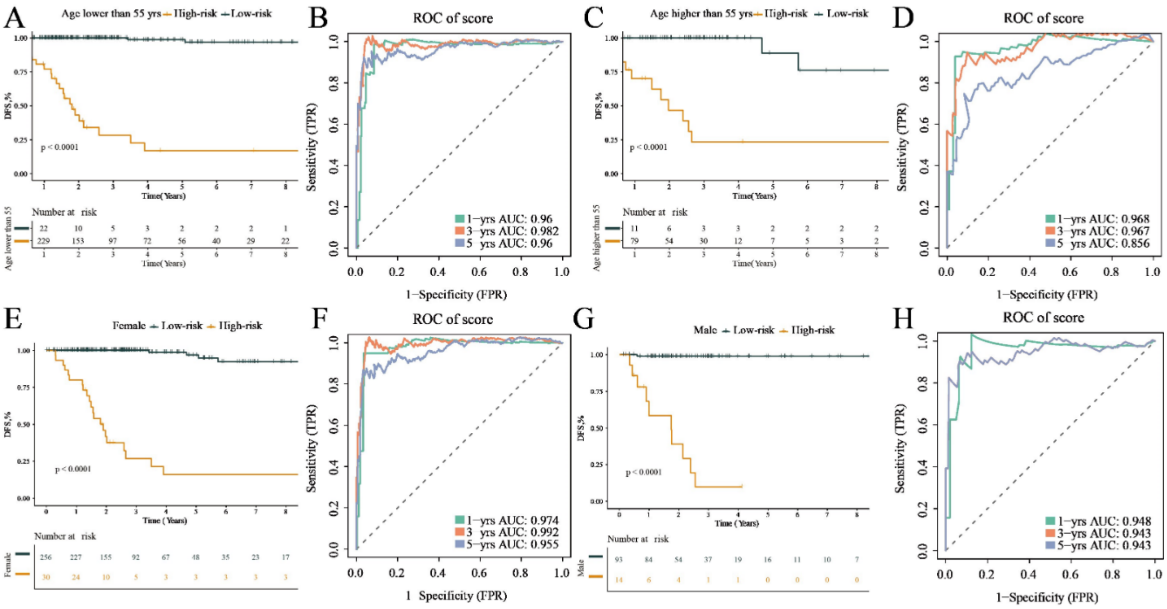

(A) KM curves of the age lower than 55 years old group. (B) ROC curves for the age lower than 55 years old group. (C) KM curves of the age higher than 55 years old group. (D) ROC curves for the age higher than 55 years old group. (E) KM curves of the female group. (F) ROC curves for the female group. (G) KM curves of the male group. (H) ROC curves for the male group.

Supplementary Figure S8 Correlation analysis to assess the association between risk scores and different stages group.

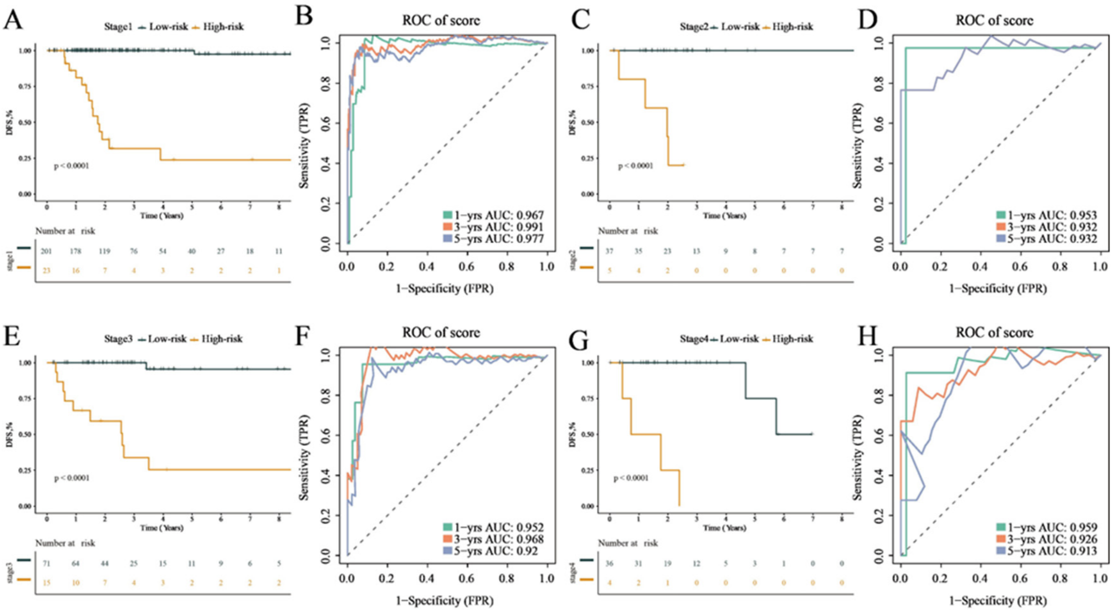

(A) KM curves of the stage 1 group. (B) ROC curves for the stage 1 group. (C) KM curves of the stage 2 group. (D) ROC curves for the stage 2 group. (E) KM curves of the stage 3 group. (F) ROC curves for the stage 3 group. (G) KM curves of the stage 4 group. (H) ROC curves for the stage 4 group.

Supplementary Figure S9 Correlation analysis to assess the association between risk scores and different T&N stages group.

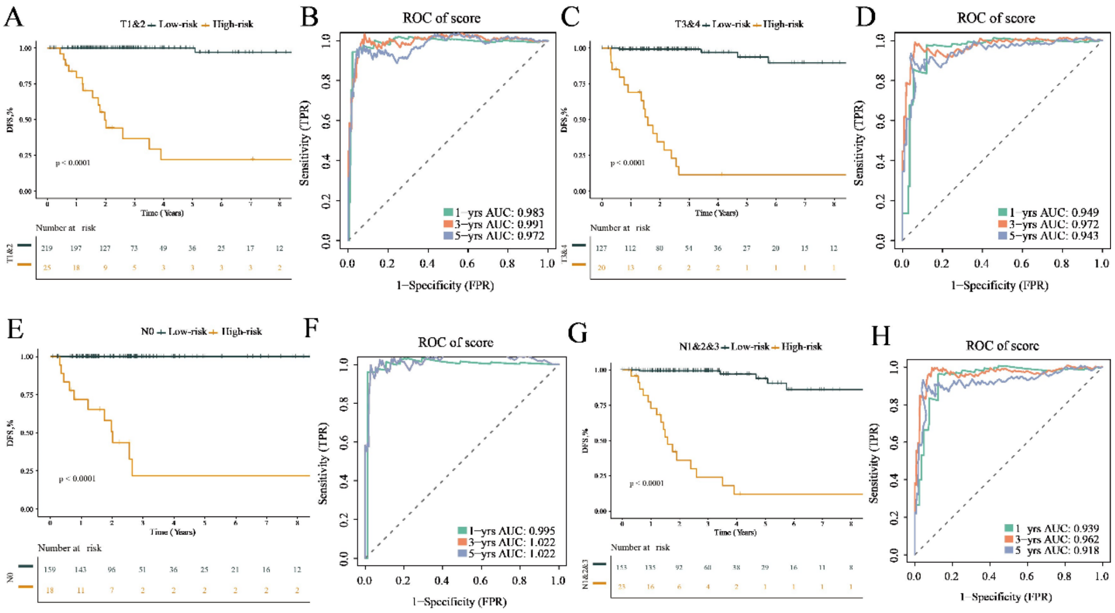

(A) KM curves of the T1&2 group. (B) ROC curves for the T1&2 group. (C) KM curves of the T3&4 group. (D) ROC curves for the T3&4 group. (E) KM curves of the N0 group. (F) ROC curves for the N0 group. (G) KM curves of the N1&2&3 group. (H) ROC curves for the N1&2&3 group.
